# Mapping and modeling human-black bear interactions in the Catskills region of New York using resource selection probability functions

**DOI:** 10.1371/journal.pone.0257716

**Published:** 2021-09-22

**Authors:** Jason S. Hagani, Sara M. Kross, Michael Clark, Rae Wynn-Grant, Mary Blair

**Affiliations:** 1 Department of Ecology, Evolution, and Environmental Biology, Columbia University, New York, New York, United States of America; 2 New York Department of Environmental Conservation Region 4, Schenectady, New York, United States of America; 3 Bren School of Environmental Science and Management, University of California Santa Barbara, Santa Barbara, California, United States of America; 4 Center for Biodiversity and Conservation, American Museum of Natural History, New York, New York, United States of America; Amity University, INDIA

## Abstract

Black bears (*Ursus americanus*) are an iconic and common species throughout much of the United States and people regularly interact with these large predators without conflict. However, negative interactions between people and bears can manifest in conflicts that can hinder conservation efforts. Black bears are highly attracted to anthropogenic sources of food, and negative interactions with people are primarily a product of trash mismanagement. In the Catskills region of New York State, home to a large population of black bears, over 400 such conflicts are reported each year. While the New York Department of Environmental Conservation (DEC) has seen progress recently in educating residents of the region on how to reduce unwanted interactions with bears, they have had less success educating the 12 million tourists that visit the Catskills each year. Understanding where conflict may occur in the future, and the environmental and anthropogenic factors that precede it, may help guide management strategies to reduce these unwanted interactions. Therefore, we designed resource selection probability functions (RSPFs) to examine the relationship between human-black bear conflicts in the Catskills with a suite of landscape and anthropogenic data, using conflicts reported to the DEC across the state of New York in 2018–2019. We found that human-black bear conflicts were more likely to occur in the residential areas of the Catskills on the urban-wildland interface; areas with relatively higher human population densities, away from dense forest, and further from heavily urbanized areas. While future work is needed to continuously validate our model predictions, our results will provide the DEC and other conservation managers in the Catskills the ability to create more targeted plans for mitigating unwanted human-black bear interactions, and provide a better understanding of the mechanisms driving human-carnivore interactions at an urban-wildland interface more generally.

## Introduction

Interactions between people and large carnivores create conflicts that are among the most common and widespread conservation challenges in the world [1]. Despite their abundance, human-carnivore conflicts can be notoriously difficult to manage, due to the ecological importance of large carnivores and their perceived or real threat to human livelihoods [2–4]. Large carnivores are essential, often keystone species in many ecosystems throughout the globe [5], and serve an important role in many cultural systems [3]. However, these same species can cause injuries to people, depredate livestock, and interact with humans in other negative ways [6, 7]. The complexities of human-carnivore interactions vary across species, land use types, and cultural norms, so finding ways to manage the social, ecological, and economic impacts conflict requires extensive local knowledge and an understanding of the underlying factors that delineate these negative interactions [2, 3, 6].

In one example of widespread human-carnivore interactions, conflicts between people and the American black bear (*Ursus americanus*) have been steadily increasing throughout the United States in recent decades [8–10]. Populations of both black bears [11, 12] and humans have grown country-wide, leading to more interactions between people and bears [13]. Black bears have a keen sense of smell and can become highly attracted to anthropogenic sources of food, especially during their period of hyperphagia before hibernation [14, 15]. As a result, black bears often enter suburban and residential areas [13], can become reliant on anthropogenic food sources, become habituated to human presence, and may damage private property [16–18]. Black bears are considered to be the least aggressive of all North American bears, and few of these interactions have resulted in injury or death to people [13]. However, in extreme cases, negative interactions between humans and black bears can lead to the euthanization of overly aggressive “problem” animals [19, 20], or injury to people. Mitigating conflicts between people and black bears in the United States is difficult due to bears’ wide distribution and adaptability, especially as urbanization and exurban developments increase [13, 21]. Therefore, finding effective solutions to reduce the number of interactions between people and black bears is critical.

**Fig 1:**
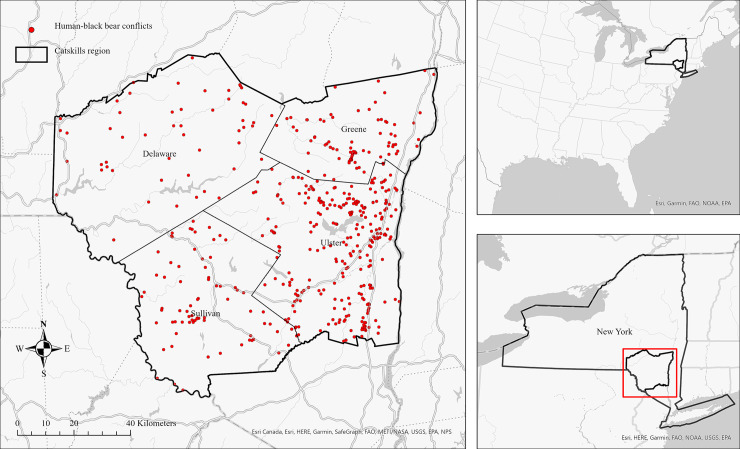
Human-black bear interactions (n = 842) recorded by the NYDEC in 2018 and 2019. Catskill counties are Greene, Delaware, Sullivan, and Ulster.

In the Catskills region of New York State (Fig 1), conflicts with black bears are a concern to local resource managers, governments, and residents. The Catskills is a mountainous region 200km north of New York City popular with both year-round residents and tourists (and the location of the famous Woodstock concerts). Currently over 2000 black bears inhabit the Catskills region—an almost tenfold increase since the 1970s [22]. This continued increase is due to successful conservation programs implemented by the New York Department of Environmental Conservation (DEC) and other wildlife organizations [23]. From 2018–2019, 842 complaints were registered with the DEC on black bears in the region (Fig 1). Recently, the DEC has created a number of management approaches to reduce human-black bear interactions. The hunting season was extended in an effort to manage growing black bear populations, with the firearm season beginning 16 days earlier in September in the Catskills and Western Hudson Valley [23]. This extension allowed for 584 Catskill black bears to be harvested during the first year of its extension (2014), compared to 451 the previous year [23, 24]. Additionally, bear-proof garbage dumpsters are becoming more widespread in some regions of the Catskills [23, 25], and the DEC continues to promote education on bear-safety in the region. Nonetheless, conflict with black bears persist.

Despite the success of the DEC in reducing unwanted interactions between black bears and residents in the Catskills, there has been less success with short-term visitors (L. Bifaro, pers. comm.). Coinciding with the recent growth of black bear numbers in the region is a substantial increase in the Catskills tourist population. Every year the Catskills region welcomes 12 million tourists [26], many of whom use rental sites like AirBnB and Vrbo to locate short-term accommodations. Thousands of short-term visitors rent the 4,376 available homes in the Catskills each year (data: AirDNA, Denver, U.S.), yet little has been done to ensure that these tourists are aware of how to properly manage trash in an area with abundant black bears. There is little precedent throughout the country on how to successfully educate transient visitors to tourist regions, which is important given that many are unaware of the dangers of black bears and some even actively attempt to attract them [27]. In other areas with long-established black bear and tourist populations, such as the Lake Tahoe region in Nevada, reducing interactions between tourists and black bears is similarly challenging [27].

While the DEC has recently engaged with efforts to create an educational program designed to inform tourists to the Catskills on bear-safe behaviors, work is needed to determine how and where to most effectively distribute it. Our study addressed this latter need by mapping and modeling human-black bear conflicts in the region. Using data on human-black bear interactions collected by the DEC throughout the state, we built Resource Selection Probability Function models (RSPFs) that examine the relationship between these interactions and a suite of landscape and anthropogenic variables. Our results will provide the DEC with a tool to effectively target areas to implement their educational program for tourists, promote a safer coexistence between an iconic Catskills species and the tourists vital to the region, and highlight a method for predicting and managing human-wildlife conflicts more broadly.

## Methods

### Study area & conflict data

Data on interactions between humans and black bears is recorded by the DEC by compiling information from their responses to complaints submitted by the public. For each recorded interaction, the DEC noted the type of complaint (livestock injury, residential damage, illegal feeding, etc.), the address, and the severity of the incident (denoted by classes 1 to 4, 1 being the most severe). We were provided with all recorded instances of human-black bear conflict in the entire state from January 1, 2018- through December 31, 2019. In that time, 2,589 conflicts occurred statewide; 842 of which were located in the Catskills. Because black bears are generalists [28, 29] and not confined to political boundaries of the Catskills, we used all incidences in New York State to build our predictive models, mapped conflict throughout the entire state, and reported our specific findings for the region. Black bears’ range in New York covers most of the state, and the species occurs throughout the entire Catskills region [30]. The Catskills region is primarily forested (82%), with pockets of grassland (9%), urban areas (4%), and cropland (2%). The region is relatively stable with regards to major changes in land use or topography.

The 2,589 locations of conflict were spatially thinned by 5km in R Studio Version 1.2.5033 [31, 32] using a randomization algorithm in the package “spThin” [33]. Thinning location data is often necessary to reduce spatial autocorrelation between points [33]; 5km was selected because of its relevance to the average black bear home range size of about 25km^2^ [34]. Additional points were excluded if their locations were obviously reported erroneously, such as those reported in Long Island and New York City where there have been no confirmed sightings of black bears in recent years. This left 683 remaining locations that could be used to build models. For the same reasoning as the thinning process, a 5km buffer was then created around each of those conflict locations in QGIS. These buffers were then subtracted from the area of New York State, and 3,415 random locations were generated within the remaining extent (> 5 for each conflict location; [34]). These random locations served as proxies for “non-conflict” locations, in order to compare regions where conflict occurred with those where it did not.

### GIS layers

Twelve environmental, landscape, and anthropogenic variables were selected to model human-black bear conflicts (Table 1). General landscape characteristics, such as elevation, aspect, terrain ruggedness, and land cover are often used when modeling species distributions or human-wildlife conflicts [34–37]. Forest density, distance to forests, and distance to streams, were selected because of their biological relevance to black bear habitat as described by previous literature [34, 38]. Lastly, anthropogenic variables (human population density, road density, distance to roads, distance to urban areas, and distance to recreational areas) were chosen to predict the distribution of human influence. Distance layers were built in ArcGIS Pro 10.6 [39] using the Euclidean Distance tool. Land cover was reclassified from its original sixteen categories to just eight: forest, urban, shrubland, grassland, barren/minimal vegetation, agriculture, wetland, and water. In order to reduce the influence of extreme outliers on our models’ outputs, distance to forests and population density data were log-transformed before analyzing. All layers were projected to NAD83 UTM Zone 18N coordinate reference system (EPSG:26918), cropped to the exact same extent (xmin, xmax, ymin, ymax = 1597585, 2345800, -307196, 350164), and resampled to a 50-meter resolution using the nearest neighbor method in R. Values at each conflict and non-conflict location were extracted from each covariate in ArcGIS Pro. It is important to note a potential temporal mismatch between our conflict data (2018–2019) and some of our GIS data (2000–2019; Table 1).

**Table 1:** Description of the environmental, landscape, and anthropogenic variables used to model human-black bear conflicts in the Catskills.

Variable	Description	Data Type	Units	Year Created	Source
Elevation	Height above sea level	Continuous	Meters	2000	WorldClim
Aspect	Compass direction of slope	Continuous	Degrees	2000	Generated in QGIS using the “Elevation” layer
Terrain ruggedness	Describes the amount of elevation change between neighboring grid cells	Continuous	Index	2000	Generated in QGIS using the “Elevation” layer
Human population density	Density of human population	Continuous	People/km	2019	LandScan
Land cover	Classification of land cover type	Categorical	N/A	2013	ESRI Living Atlas
Road density	Density of roads	Continuous	Km of road/km raster cell	2014	ESRI Living Atlas
Distance to roads*	Straight line distance to major roads	Continuous	Meters	2019	US Census TIGER Shapefiles
Distance to* streams	Straight line distance to streams	Continuous	Meters	2014	NY DEC
Distance to urban areas*	Straight line distance to urban areas (human population density > 500k)	Continuous	Meters	2010	ESRI Living Atlas
Distance to recreational areas*	Straight line distance to recreational areas (parks, campsites, picnic areas, etc.)	Continuous	Meters	N/A	NY DEC
Distance to forest cover*	Straight line distance to forest cover	Continuous	Meters	2016	ESRI Living Atlas
Forest density (canopy cover)	Proportion of each pixel covered by tree canopy	Continuous	Percent	2016	ESRI Living Atlas

* denotes layers created in ArcGIS using the Euclidean Distance tool.

### Model selection and data analysis

Resource Selection Probability Functions (RSPFs) are commonly used to model large carnivores’ distribution, movements, mortality, and interactions with people [34, 40–42]. RSPFs operate under the assumption that certain portions of a landscape are used disproportionately [43]. RSPFs generally adhere to the following equation [34, 40]:

$$w(x)=\frac{exp\;(\beta_{0}+\beta_{1}x_{1}+\ldots \beta_{n}x_{n})}{1+exp\;(\beta_{0}+\beta_{1}x_{1}+\ldots \beta_{n}x_{n})}$$ Eq 1

where w*(*x*) equals the probability of conflict at a given location, *x* refers to the set of covariates used, and β are the parameters of the model.

We generated RSPFs in R Studio using the package “Resource Selection” [43]. We set “m”, which defines the matching of conflict and non-conflict points, to 0 and “B”, which sets the number of bootstrap iterations to run, to 1000 [43]. Before creating models, we assessed collinearity between covariates based on the inspection of variance inflation factors (VIFs); variables with a VIF above 10 were removed from the model [44, 45]. None of the variables met this criterion for collinearity, and therefore all were retained. We also examined correlation between covariates by building a correlation matrix; for covariates that shared an R^2^ coefficient greater than 0.7 [46], the one hypothesized to be more relevant to black bears or conflict would be retained. No two variables shared an R^2^ that exceed this threshold; therefore, all were considered. We examined all possible models derived from a combination of our twelve covariates using the “MuMIn” package in R [47]. We selected the model with the lowest AIC value [48]. For models with comparative AICs (ΔAIC > 2.0), the one with the fewer terms was selected [49].

### Model validation

To justify our use of data from throughout New York State to make predictions in the Catskills, we examined the predictive power of our model by spatially subsetting our data in two ways. First, we used data from outside of the Catskills region to train our model (conflicts = 543, non-conflicts = 3159), and data from within the region to test it (conflicts = 140, non-conflicts = 256). We also used k-fold cross-validation across the entire state-wide dataset to determine overall predictive power. We evaluated the predictive ability of our models by calculating area-under-the-curve (AUC) values and omission error. The AUC measures the area under the receiver operator curve (ROC), and provides a value between 0 and 1 which describes the predictive power of a model [50]. A model with an AUC of 0.5 is no better than a random guess, and a model with an AUC of 1.0 is completely predictive [50]. Typically, models with an AUC over 0.7 or 0.8 are considered to be robust [50]. Omission error describes the proportion of conflict and non-conflict locations that were incorrectly identified by our model based on a certain threshold; we chose to use an equal sensitivity and specificity threshold [51]. If our statewide model was deemed sufficiently representative of the Catskills (AUC > 0.7 and overall omission error < 0.3), we then applied k-fold cross-validation to test the predictive power of the most parsimonious model [52, 53]. We subsetted the statewide data into five random k-fold groups; four were used to train the model and one was used to test it. This process was repeated four times, to ensure that each group was used as the testing dataset once. If cross-validation yielded models with a high predictive power (AUC > 0.7 and overall omission error < 0.3), we then retrained a model with all data and projected its outputs as a map of conflict hotspots throughout New York State. We created this map using the “Raster” package [54] in R Studio, cropped it to the extent of the Catskills, then refined it in ArcGIS Pro for improved visualization.

## Results

### Human-black bear conflicts

**Fig 2:**
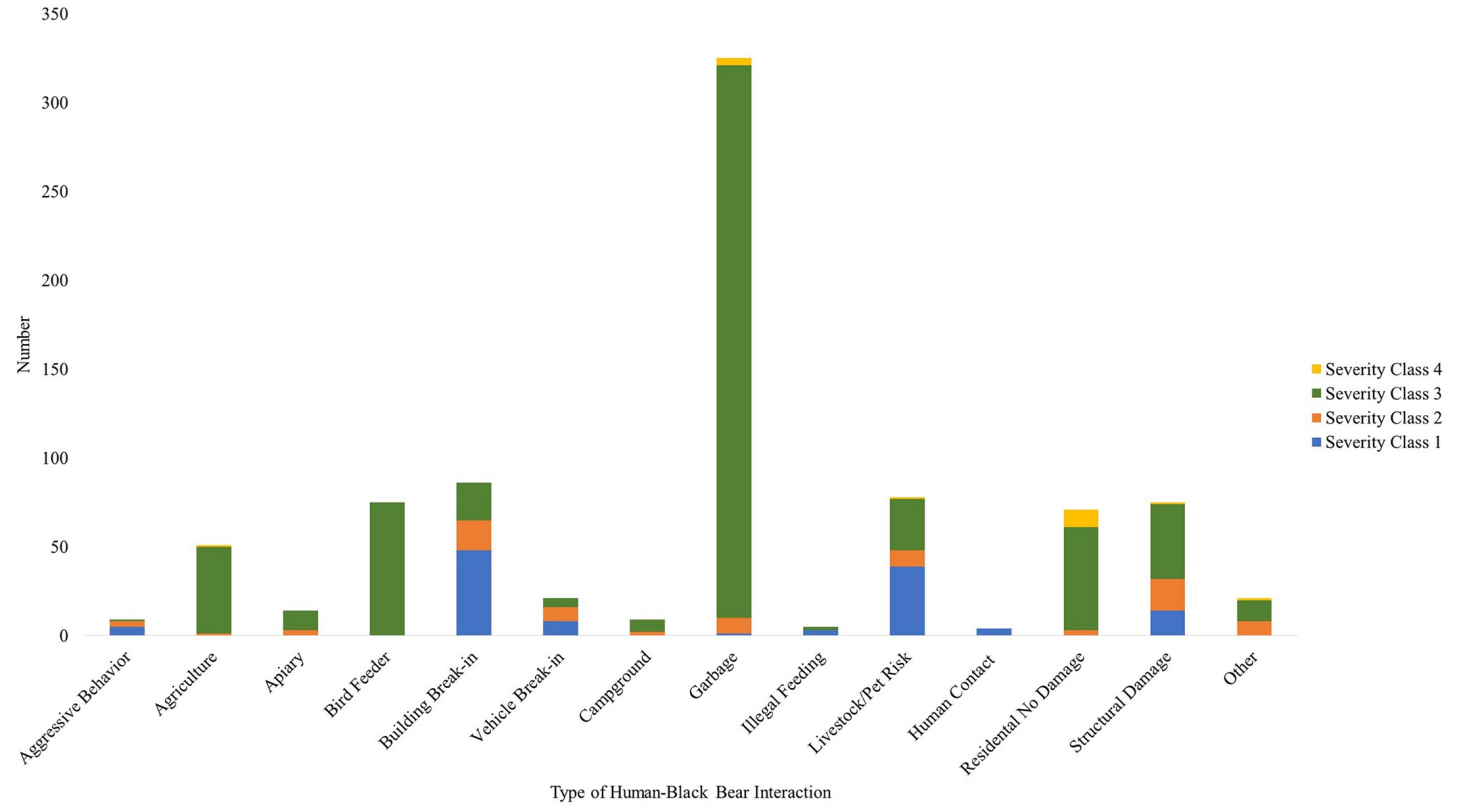
Catskills’ human-black bear interactions recorded by the DEC in 2018 and 2019 (n = 842). Bars are color coded by severity class; 1 (blue) is the highest severity, 4 (yellow) the lowest.

Of the 842 conflicts recorded by the DEC in 2018 and 2019 in the Catskills region, 39% (325) of them were classified as garbage break-ins (Fig 2). Contact with humans (4 occurrences), illegal feeding (5 occurrences) and campground issues (9 occurrences) were the rarest types of interactions (Fig 2). Based on the DEC’s severity class system, in which 1 is an interaction of the highest severity and 4 of the least, 15% were highly severe (1), 84% were mildly severe (2–3), and 1% were not severe at all (4).

### Model outputs

The most parsimonious model included seven covariates: elevation, distance to forests, land cover, human population density, distance to recreational areas, road density, and distance to urban areas (Table 2). Our parsimonious model determined that all continuous covariates, with the exception of road density (p = 0.828), were significantly correlated with the probability of human-black bear conflicts (Table 3). All significant continuous covariates were positively correlated with conflict, suggesting that as they increased so did the probability of any conflict occurring. Human-black bear conflict was also significantly and negatively correlated with a variety of land cover classes including forests (p < 0.001) and urban areas (p < 0.001).

**Table 2:** The five “best” RSPF models, based on AIC scores.

#	Model	df	AIC	ΔAIC
**1**	**elevation + land cover + distance to forests + population density + distance to recreational areas + road density + distance to urban areas**	**14**	**10283.4**	**0.0**
2	elevation + forest density + land cover + distance to forests + population density + distance to recreational areas + road density + distance to urban areas	15	10284.9	1.5
3	elevation + land cover + distance to forests + population density + distance to recreational areas + distance to streams + terrain ruggedness + distance to urban areas	15	10288.4	5.0
4	elevation + forest density + land cover + distance to forests + population density + distance to recreational areas + distance to streams + distance to urban areas	15	10289.0	5.6
5	elevation + land cover + distance to forests + population density + distance to streams + terrain ruggedness + distance to urban areas	14	10289.7	6.3

The most parsimonious model is bolded. Distance to forests and population density were log-transformed.

**Table 3:** Coefficient estimates for each parameter and its corresponding significance based on the optimal resource selection probability function model.

Covariate	Estimate	SE	p-value
Elevation	2.65 x 10^−3^	4.00 x 10^−4^	< 0.001
Distance to forests	0.022	2.76 x 10^−5^	< 0.001
Population density	0.774	0.083	< 0.001
Distance to recreational areas	1.59 x 10^−5^	5.22 x 10^−6^	0.002
Road density	0.014	0.068	0.828
Distance to urban areas	4.36 x 10^−5^	8.64 x 10^−6^	< 0.001

Estimates are not provided for the categorical land cover covariate.

### Model validation

**Fig 3:**
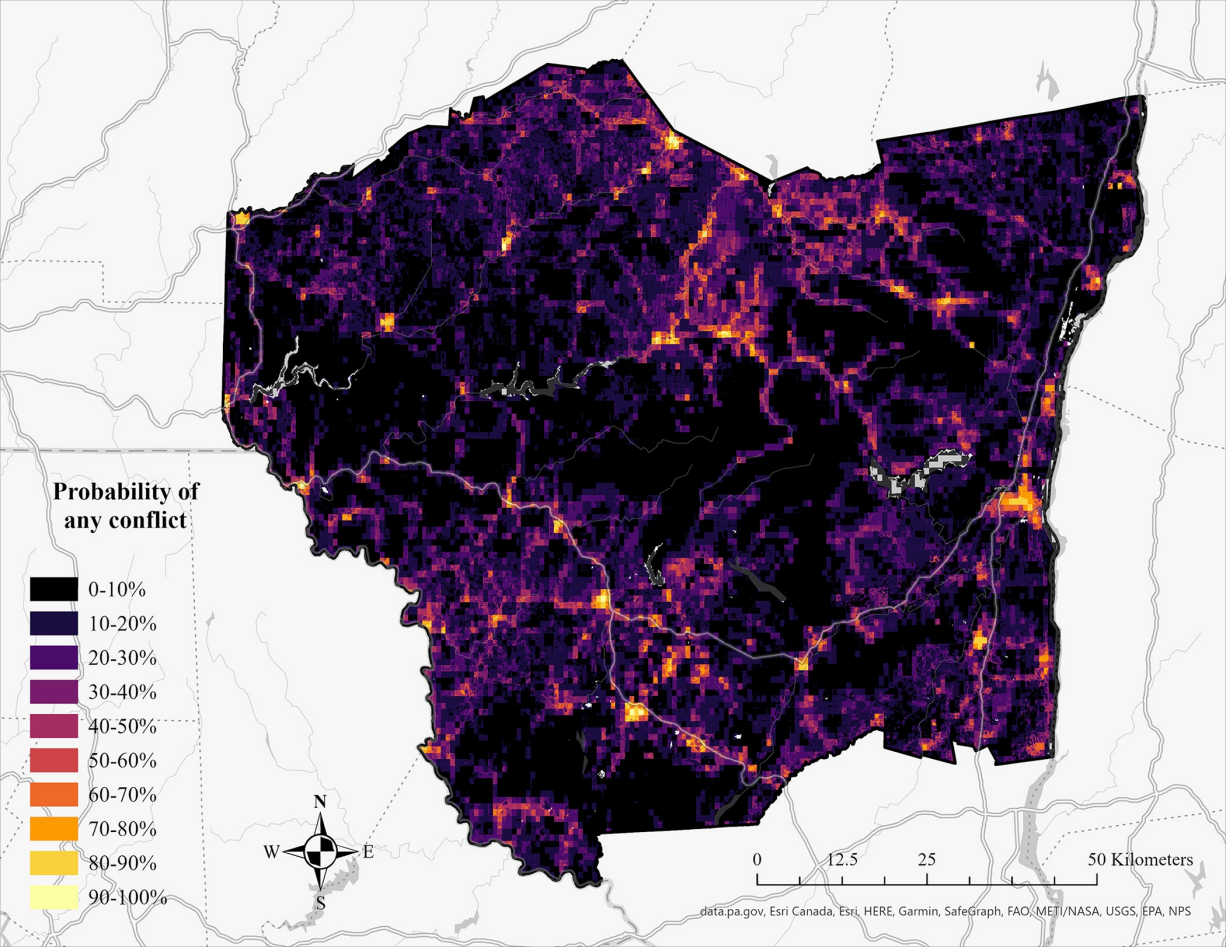
Map depicting risk human-black bear conflicts in the Catskills region of New York. Map is based on our optimal RSPF model. Brighter, warmer colors indicate a higher probability of conflict, while darker, cooler colors represent low-risk areas of conflict.

We determined the ability of our statewide model to accurately represent the Catskills region by using data from outside the Catskills to train the model, and data from within the region to test the model. This test yielded an AUC value of 0.801, and an overall omission error of 0.291 (0.326 for conflict locations, 0.254 for non-conflict locations). Using k-fold cross validation across the state-wide dataset generated models that varied in AUC, AIC, and omission error (Table 4). All data were then recompiled, and the model produced was then projected to map human-black bear interactions in the region (AIC = 10283.4, AUC = 0.795, overall emission error = 0.234; Fig 3).

**Table 4:** Results of k-fold cross validation.

K-fold	AIC	AUC	Omission Error (Conflicts)	Omission Error (Non-Conflicts)	Overall Omission Error
1	8021.82	0.808	0.300	0.336	0.247
2	7966.14	0.749	0.422	0.156	0.200
3	8027.70	0.815	0.265	0.272	0.271
4	8004.01	0.777	0.315	0.237	0.250
5	7989.81	0.782	0.182	0.365	0.334

## Discussion

As black bear populations rebound in many areas alongside the simultaneous growth of human residential developments, the challenges of mitigating human-bear conflict are growing [13, 21]. Our model reinforces the presumption that anthropogenic presence was the driver of recent conflicts in New York State; distance to recreational areas, distance to urban areas, and human population density were each significantly correlated with occurrence of human-black bear conflicts. The model, created to identify potential hotspots of black bear-human conflict in the Catskills, had good predictive power and will be helpful for DEC biologists to identify areas where future tourist education is most needed. We recommend that, even in the Catskills where conflict is relatively common, the DEC prioritize outreach efforts on areas of higher residential density and at higher elevations. Our model described the probability of conflict as increasing further from urban areas and recreational areas, but in areas of higher human population densities. This is a reasonable reflection of both bear- and human- behaviors: black bears rarely inhabit highly urbanized areas and fewer humans frequent the natural regions that comprise recreational areas. In the Catskills, these results suggest that the potential for human-black bear interactions is likely at its highest in moderately populated areas that are adjacent to public lands, consistent with research in other regions of New York [55] or on black bears in other parts of the U.S. [56, 57]. This expected parabolic relationship between anthropogenic variables and human-wildlife interactions has been described previously in human-wolf conflicts in Michigan [58]. Given the abundance of trash-related incidents in the Catskills region each year, human-bear interactions will likely be highest in residential, suburban areas. However, it is important to note that our results include the likelihood of *any* conflict with black bears occurring, and was based on conflict reports over a two-year time frame, so they do not predict the frequency or severity of conflict at any given point. This caveat may suggest that our model is likely an underestimate of the true level of human-black bear conflict in the region.

Our model provides valuable information on how human-black bear conflicts overlap with black bear habitat. Black bears are generalists, and are able to live in a variety of ecosystems and landscapes [28, 29]. Our model predicts that human-bear interactions were more likely to occur at higher elevations. Across the state of New York, most highly urbanized areas are located at lower elevations, while higher elevations include the mountainous regions, such as the Catskills, that are popular with tourists and for scenic vacation homes. While our model also suggested that the probability of a human-black bear interaction was highest further from forests, this result was likely driven by the dense, heavily-forested areas that comprise large swaths of the more isolated regions of the state. It is imperative to recognize that this conflict map was built using data on bear conflicts from the entire state of New York, and that conflict was modeled statewide. Including data from the entire state, not just the Catskills region, is necessary because wildlife rarely adhere to political boundaries, but lowers the specificity of our model results. Black bears often move in and out of the Catskills region—therefore these boundaries cannot be used to constrain models too tightly. While our model did exceed the minimum AUC threshold required [50], it is likely that human-black bear conflict in other parts of the state influenced our predictions. Expanding the models to consider a more ecologically relevant study extent, rather than using geographic boundaries, will improve the modeling process but requires data from multiple state wildlife agencies.

In general, these findings further support the idea that human-black bear conflicts are expected to occur at the urban-wildland interface, rather than in highly urbanized or wild areas [55–57]. As such, we expect tourists staying in suburban areas at high elevations to be at the highest risk of interacting with black bears. Targeting these areas for implementing an educational program may therefore prove most effective. However, human-wildlife interactions are complex, especially those involving carnivores. Understanding how to manage potential conflicts requires incorporating not just ecological knowledge, but social factors as well [6]. Importantly, our underlying data relies on complaint calls to the DEC, which are likely influenced by individuals’ perceptions of conflict which is influenced by demographic and social variables; [59–63], and their knowledge of when and how to report interactions. We recommend that wildlife agencies collecting reports of human-wildlife conflicts also collect demographic data, so such data may be incorporated into future modeling efforts. Furthermore, tourists like those that visit the Catskills may be especially accepting of black bears—as wildlife is one of the main draws to the region (L. Bifaro pers. comm.). This acceptance may lead to interactions that are harmful for black bears, but thrilling for tourists. Similarly, regions with highly educated populations on bear safety may have lower rates of conflict than expected given our model’s projections. Balancing all of these varying perspectives is a necessary challenge when interpreting predictive models like ours. Therefore, applying maps and models of human-wildlife interactions to actual management will require incorporating an expert understanding of the social dynamics in the region of interest. In general, managing human-carnivore conflicts also often requires very localized knowledge and management [6]. However, our results may yield insight into patterns of human-black bear interactions more broadly, especially in regions similar to the Catskills with a large tourist population and urban and natural areas in close proximity.

Future work is necessary to refine and validate our model’s projections. Our model was able to differentiate between conflict and non-conflict locations with a high degree of statistical accuracy. However, the complexity of reality can never be completely captured. We hope our model will be used in conjunction with expert knowledge, field surveys, and the already established methods for reducing conflict employed by the DEC, and it should not be considered a perfect predictor of future conflict. Previous studies mapping and modeling human-carnivore conflicts have validated their results with great success. Treves and Rabenhorst (2017) [64], for example, showed that their model of human-wolf interactions in Wisconsin accurately predicted 91% of conflicts the three years following its creation. Similar research conducted in Idaho was able to classify 84% of future wolf-livestock depredations in areas designated as “high” or “very high” risk for conflict [65].

Human-carnivore conflicts are a persistent, pervasive problem for both wildlife conservation and human wellbeing. However, wildlife managers often have limited resources to address human-wildlife conflict issues [66]. Furthermore, areas popular with tourists and the continuing expansion of private home-rental services can present additional challenges for implementing effective education to mitigate conflict, especially when wildlife agencies are already stretched thin. The use of predictive conflict models is one method for targeting outreach and education in areas where conflict is most likely to occur. Our model was the first step in that direction, and shows promise for these methods prioritizing outreach.

## Supporting information

S1 File Dataset containing the values used to generate resource selection probability function models (RSPFs), excluding location data. Dataset includes the following potential covariates: elevation. The dependent variable (“Conflict”) is binary; “1” denotes a human-black bear conflict, “0” denotes a randomly generated non-conflict location. See Table 1 for more information about each covariate.
(CSV)

S1 Table Regression outputs for the most parsimonious resource selection probability function model (RSPF) that was used to create a map of human-black bear conflict hotspots. (DOCX)
