# Supplementary material for: Mapping and modeling human-black bear interactions in the Catskills region of New York using resource selection probability functions
Source: PLoS One. 2021 Sep 22;16(9):e0257716. doi: 10.1371/journal.pone.0257716 (PMC8457482; doi:10.1371/journal.pone.0257716)
Supplement: S1 Table — (DOCX) [file pone.0257716.s002.docx]

**lRSPF most parsimonious model outputs:**

AIC = 10283.4, average AUC = 0.786, average omission error = 0.260

| **Covariate** | **Estimate** | **Standard Error** | **p-value** | **95% CI** |
| --- | --- | --- | --- | --- |
| Elevation | 2.65 x 10^-3^ | 4.00 x 10^-4^ | < 0.001* | 1.87 x 10^-3^ –  3.43 x 10^-3^ |
| Distance to forests | 0.02 | 2.76 x 10^-5^ | < 0.001 * | 2.19 x 10^-2^ –  2.21 x 10^-2^ |
| Population density | 0.77 | 0.08 | < 0.001* | 0.61 – 0.94 |
| Distance to recreational areas | 1.59 x 10^-5^ | 5.22 x 10^-6^ | 0.002* | 5.67 x 10^-6^ –  2.61 x 10^-5^ |
| Road density | 0.01 | 0.07 | 0.828 | -0.12 – 0.15 |
| Distance to urban areas | 4.36 x 10^-5^ | 8.64 x 10^-6^ | < 0.001* | 2.67 x 10^-5^ –  6.05 x 10^-5^ |
| Land cover 1 (forests) | -5.27 | 0.32 | < 0.001* | -5.90 – -4.65 |
| Land cover 2 (urban) | -4.98 | 0.51 | < 0.001* | -5.98 – -3.98 |
| Land cover 3 (shrubland) | -6.47 | 0.99 | < 0.001* | -8.41 – -4.53 |
| Land cover 4 (grassland) | -6.02 | 0.34 | < 0.001* | -6.69 – -5.35 |
| Land cover 5 (minimal vegetation) | -5.79 | 2.47 | 0.019* | -10.63 – -0.95 |
| Land cover 7 (agriculture) | -6.55 | 0.34 | < 0.001* | -7.22 – -5.88 |
| Land cover 9 (wetland) | -5.91 | 1.02 | < 0.001* | -7.91 – -3.91 |
| Land cover 11 (water/ice/snow) | -6.11 | 0.42 | < 0.001* | -6.93 – -5.29 |
